# Ischial Tuberosity Avulsion Fracture Mimicking Calcified Mass on Plain Films: A Case Report

**DOI:** 10.7759/cureus.53165

**Published:** 2024-01-29

**Authors:** Mason A Williams, Lena Naffaa

**Affiliations:** 1 Radiology, University of Central Florida College of Medicine, Orlando, USA; 2 Radiology, Nemours Children's Health, Orlando, USA

**Keywords:** avulsion, pediatric radiology, sports related injuries, mri pelvis, calcified mass, ischial tuberosity, avulsion fracture

## Abstract

Ischial tuberosity avulsion fractures are overall uncommon but are known injuries in the adolescent population. They are the result of sudden, forceful contraction of the hamstring muscle groups. The characteristic radiographic appearance of an ischial tuberosity avulsion fracture is of an irregular ischial margin and a nearby avulsed bone fragment. Callous formation may ensue and appears as a calcific density in the region of injury. Awareness of the spectrum of radiographic presentations can help ensure correct diagnosis and minimize concern for alternative underlying diagnoses. This case report describes a 14-year-old boy with a chronic ischial tuberosity avulsion fracture which demonstrated an unusual presentation on radiographs and required MRI to confirm the diagnosis and rule out other potentially ominous pathology.

## Introduction

The large and powerful hamstrings and adductor muscle groups insert at the ischial tuberosity. The most common site of avulsion fractures in adolescents is in the lower extremities, including at the ischial tuberosity [1]. The mechanism of injury is sudden, forceful muscle contraction [1]. Avulsion fractures at this location result in portions of the ischium avulsed and held within surrounding soft tissues, often with associated ischial tuberosity margin irregularity and similar irregularity within the avulsed fracture piece [2]. Treatment is generally non-surgical, and patients are managed conservatively. Adherence to activity restrictions is a salient part of management to allow healing and prevent secondary injury.

## Case presentation

**Figure 1:**
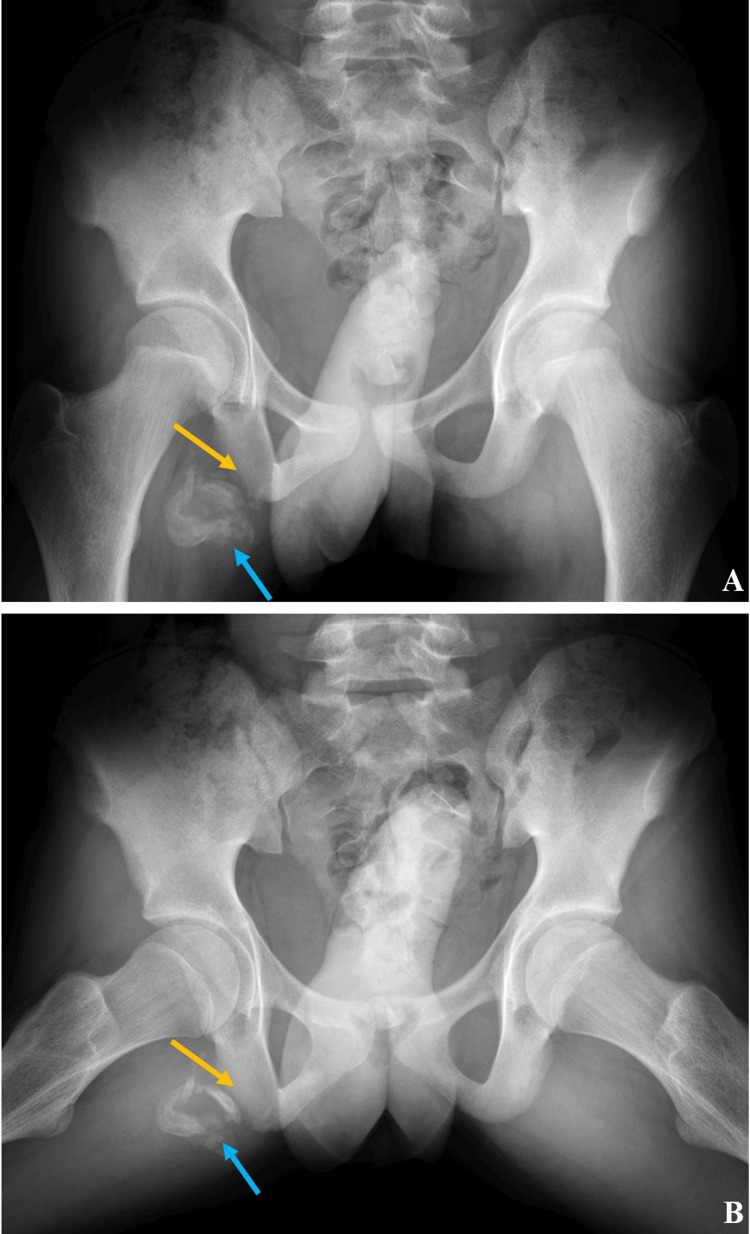
Radiographs of bilateral hips AP (Figure 1A) and frog leg (Figure 1B) radiographs demonstrate lucency and irregularity of right ischial tuberosity suggesting avulsion fracture (yellow arrows). There is a cluster of well-corticated, ovoid, and crescent-shaped calcifications in the soft tissues near the dominant fracture fragment (blue arrows).

The patient is a 14-year-old boy with no significant past medical history who presented to the orthopedic department of a children’s hospital for evaluation of a right hip pain. The patient reported that he fell down two months prior to the visit while playing football and heard a popping sound followed by pain in the posterior aspect of his right hip. The patient did not seek medical care at the time of injury and continued participating in physical activities like water skiing. As the pain did not resolve, he was then referred to orthopedics. Upon presentation to the orthopedic department, the patient described ongoing pain localized to the right ischial tuberosity and was associated with tingling down the posterior leg to the level of the mid-thigh. It was exacerbated by activity and improved with rest. There were no associated symptoms and no accompanying bruising. The severity of pain at the time of evaluation was as high as 4/10. The physical exam was significant only for decreased strength (3/5) with resisted knee flexion. The patient had not received imaging or treatment prior to the visit. Frontal and frog-leg radiographs (Figure 1A, 1B) of the pelvis were ordered for assessment.

**Figure 2:**
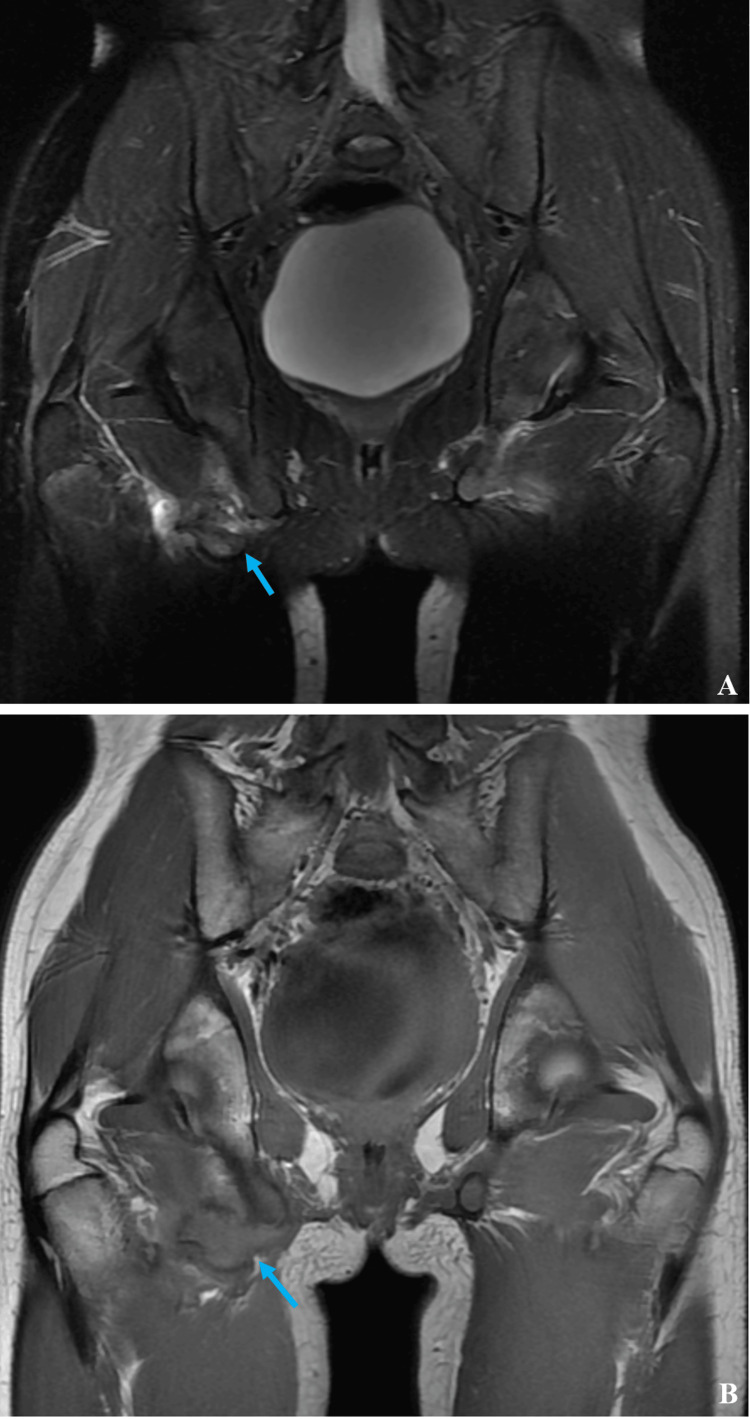
Magnetic resonance images of patient hips Coronal T2-weighted image with fat saturation (A) and coronal T1-weighted image (B) demonstrate that the cluster of ovoid calcifications adjacent to the avulsed right ischial tuberosity fragment corresponds to several foci of callous formation in the soft tissues without a true mass (blue arrows).

Radiographs revealed components of a healing right-sided ischial tuberosity avulsion fracture and a 5.8cm cluster of well-corticated, ovoid and crescent-shaped calcifications in the soft tissues near the ischial tuberosity and adjacent to the avulsed bone fragment. The affected ischial tuberosity displayed lucency and irregular margins. The unusual appearance of clustered, irregular calcific densities in the soft tissues in conjunction with the clinical context of persistent pain two months after injury raised concern for underlying calcified soft tissue mass. Evaluation with Magnetic Resonance Imaging (MRI) was warranted for further characterization (Figure 2A, 2B).

MRI demonstrated a dark signal on both T1 and T2 weighted images suggesting calcifications. The appearance on MRI confirmed the diagnosis of ischial tuberosity avulsion fracture, and the area of concern in the adjacent soft tissues was due to the accumulation of callous adjacent to the originally avulsed bone fragment.

The patient was expected to make a full recovery with conservative management including rest, physical therapy, and progressive return to activity. The patient’s right ischial tuberosity avulsion fracture improved clinically over several months and he was able to make a gradual return to his usual activities and sports.

## Discussion

Ischial tuberosity avulsion fractures are an overall uncommon but known injury pattern in adolescents, particularly those engaging in sports and physical activity [1]. The avulsion injury generally occurs at the apophysis, a known point of weakness. Avulsion fractures often display a typical appearance inclusive of an irregular ischium margin and an avulsed bone fragment in the nearby soft tissues. Chronic-type avulsion injuries can result in heterotopic bone or callous formation in the soft tissues and can mimic a more sinister process such as osteomyelitis or malignancy [2,3]. The calcified density in our patient’s case likely reflects a chronic process.

The novelty of this patient case is the unusual presentation of a characteristic phenomenon on radiographs. The calcified density in the soft tissues appears as well-corticated, ovoid, and crescent-shaped calcifications with nearby lucency of the ischial tuberosity as seen in Figure 1. A typical presentation of an ischial tuberosity avulsion fracture is a fracture segment that is crescent-shaped or shell-shaped. In the acute setting, the fragment shape often resembles the parent bone such that the two pieces’ shapes appear that they could be put back together like a puzzle. In the chronic setting, repetitive callous formations may be present which can mimic multiple ossified fragments of different shapes and sizes that may not appear to fit back together like a puzzle. The unusual appearance of the fragments and callous in this case requires consideration of alternative diagnoses, such as soft tissue mass. Soft tissue masses to be considered include benign masses that might also show calcific foci such as vascular malformation, hematoma, fat necrosis, and lipoma [4-6]. Moreover, concerning lesions such as lymphoma or a sarcoma variant, such as spindle cell, fibrosarcoma, rhabdomyosarcoma, or Ewing must also be considered [4-6].

The concern for a potentially more ominous pathology warrants MRI follow-up in cases such as ours [7]. MRI confirmation of avulsion fracture with callous formation allows this case to add to the spectrum of imaging presentations of ischial tuberosity avulsion fracture. Awareness of this unusual presentation can help confirm the diagnosis on imaging and mitigate concern for alternative benign or malignant pathologies.

## Conclusions

Ischial tuberosity avulsion fractures are a known injury pattern in adolescents resulting from forceful or sudden muscle contraction. The appearance on radiographs characteristically is an irregular ischial tuberosity margin with a nearby fracture fragment. Callous formation around the avulsed fragment may also occur and appear as a calcified density on radiographs, particularly in a chronic setting. Differentiating callous formation from an otherwise suspicious calcified soft tissue mass is paramount for clinical care. This patient case adds to the spectrum of radiographic presentations for an avulsion fracture with callous formation.
